# Retraction of: Overcoming the inadaptability of sparse group lasso for data with various group structures by stacking

**DOI:** 10.1093/bioinformatics/btad343

**Published:** 2023-06-08

Retraction of: Overcoming the inadaptability of sparse group lasso for data with various group structures by stacking, *Bioinformatics*, Volume 38, Issue 6, March 2022, Pages 1542–1549, https://doi.org/10.1093/bioinformatics/btab848 

Following article publication, the editors published an Expression of Concern about textual similarities that had been brought to their attention. The editors and authors have since been unable to resolve these issues, so the Editors-in-Chief are now retracting the article.

